# Consequences of recurrent hypoglycaemia on brain function in diabetes

**DOI:** 10.1007/s00125-020-05369-0

**Published:** 2021-03-18

**Authors:** Rory J. McCrimmon

**Affiliations:** Systems Medicine, School of Medicine, University of Dundee, Ninewells Hospital and Medical School, Dundee, UK

**Keywords:** Glucose-sensing, Glycaemic variability, Hypoglycaemia, Impaired hypoglycaemia awareness, Insulin, Oxidative stress, Review, Type 1 diabetes, Type 2 diabetes

## Abstract

The discovery of insulin and its subsequent mass manufacture transformed the lives of people with type 1 and 2 diabetes. Insulin, however, was a drug with a ‘dark side’. It brought with it the risk of iatrogenic hypoglycaemia. In this short review, the cellular consequences of recurrent hypoglycaemia, with a particular focus on the brain, are discussed. Using the ventromedial hypothalamus as an exemplar, this review highlights how recurrent hypoglycaemia has an impact on the specialised cells in the brain that are critical to the regulation of glucose homeostasis and the counterregulatory response to hypoglycaemia. In these cells, recurrent hypoglycaemia initiates a series of adaptations that ensure that they are more resilient to subsequent hypoglycaemia, but this leads to impaired hypoglycaemia awareness and a paradoxical increased risk of severe hypoglycaemia. This review also highlights how hypoglycaemia, as an oxidative stressor, may also exacerbate chronic hyperglycaemia-induced increases in oxidative stress and inflammation, leading to damage to vulnerable brain regions (and other end organs) and accelerating cognitive decline. Pre-clinical research indicates that glucose recovery following hypoglycaemia is considered a period where reactive oxygen species generation and oxidative stress are pronounced and can exacerbate the longer-term consequence of chronic hypoglycaemia. It is proposed that prior glycaemic control, hypoglycaemia and the degree of rebound hyperglycaemia interact synergistically to accelerate oxidative stress and inflammation, which may explain why increased glycaemic variability is now increasingly considered a risk factor for the complications of diabetes.

**Supplementary Information:**

The online version contains a slideset of the figures for download, which is available to authorised users, available at 10.1007/s00125-020-05369-0.

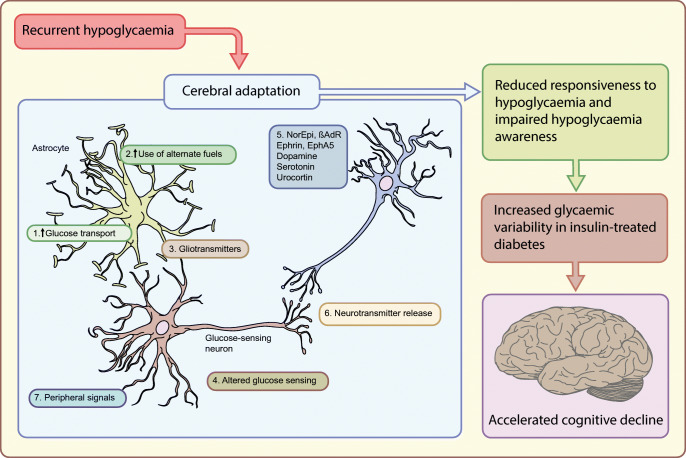

## Introduction

This year is the centenary of the discovery of insulin by Frederick Banting and Charles Best in Professor John Macleod’s department in Toronto (ON, Canada) in the summer of 1921. There can be little doubt about the impact of their discovery, which has transformed the lives of millions of people with both type 1 and 2 diabetes in the 100 years since. At the same time, it was soon discovered that insulin therapy was not without risk. Physicians reported that exogenous insulin, when delivered in excess, led to a low blood glucose; the ‘hypoglycaemic reaction’. It was also soon apparent that repeated exposure to low glucose led to, ‘reactions [that] differ so much from the original ones that patients became dangerously unaware of their onset’ [1].

Glucose homeostasis is fundamental to survival in most vertebrate species. As such, we have evolved a number of counterregulatory mechanisms designed to restore glucose homeostasis when glucose levels fall below the normal range. Over the last few decades, we have learnt that, in humans, there exists an integrated network of specialised glucose-sensing cells, found in certain key parts of the brain and in the periphery, that are able to monitor and respond to prevailing glucose levels, as well as integrate glucose homeostasis with other aspects of whole-body energy status [2, 3]. We also recognise that, in response to recurrent hypoglycaemia, these specialised glucose-sensing cells adapt, leading (through mechanisms still not entirely worked out) to a clinical syndrome called impaired awareness of hypoglycaemia. Moreover, there is increasing evidence that, in addition to making individuals susceptible to severe hypoglycaemia, these adaptations may also have consequences in terms of end-organ disease. In this short review, I will briefly discuss the cellular consequences of hypoglycaemia, focusing on the impact of recurrent hypoglycaemia in the brain. This will be illustrated by outlining the ways in which recurrent hypoglycaemia affects cells in glucose-sensing regions of the brain (leading to impaired awareness of hypoglycaemia and severe hypoglycaemia), as well as how recurrent hypoglycaemia may affect other brain regions, potentially amplifying the tissue damage that results from chronic hyperglycaemia.

## Bringing light to the dark side of insulin

Dr Philip Cryer used his 1994 Banting Lecture to the American Diabetes Association to introduce the concept of hypoglycaemia-associated autonomic failure (HAAF). Cryer outlined a series of seminal physiological studies that described how, in humans, antecedent iatrogenic hypoglycaemia resulted in defective glucose counterregulation (through reducing catecholaminergic responses to a given level of subsequent hypoglycaemia) and impaired awareness of hypoglycaemia (by reducing sympathoadrenal and symptom responses to a given level of subsequent hypoglycaemia), thereby setting up a vicious cycle whereby hypoglycaemia begets hypoglycaemia [4]. Subsequently, Dr Robert Sherwin, in his 2007 Banting Lecture, ‘Bringing light to the dark side of insulin: a journey across the blood-brain barrier’, described over three decades of research in which, using human and animal models, he and his research group had done much to help us understand how the brain detects and responds to hypoglycaemia [5].

Using the ventromedial hypothalamus (VMH) as an exemplar for a glucose-sensing region, Sherwin proposed that glucose-sensing neurons operated in a way that appeared to parallel the pancreatic islet [5]. In this model, like pancreatic beta and alpha cells, neurons in the brain respond to blood glucose levels. More specifically, glucose-excited and glucose-inhibited neurons respond to high and low blood glucose levels, respectively, in a coordinated manner. The key steps in the process of transducing the glucose signal to an alteration in neural firing rates involve sulfonylurea receptor 1 (SUR1), ATP-sensitive potassium (K_ATP_) channels, glucokinase and AMP-activated protein kinase (AMPK) [5]. Glucokinase is a critical component of this signalling mechanism because its activity is proportional to glucose concentrations. As glucose rises, therefore, so does glucokinase activity, ultimately leading to an increased ATP:AMP ratio. Supporting the role for glucokinase in glucose sensing in the brain, mice and humans with reduced glucokinase activity show an exaggerated response to hypoglycaemia [6], while glucokinase activation in hypothalamic glucose-excited neurons reverses the hyperpolarising effect of low glucose [7]. Subsequently, the increased ATP:AMP ratio with increased glucokinase activity results in closure of K_ATP_ channels, depolarising the neurons and increasing their firing rate [8]. A role for K_ATP_ channels in brain glucose sensing has been demonstrated in cells, hypothalamic slice preparations, transgenic mice, in vivo studies in rats [9], and humans with type 1 diabetes and impaired awareness of hypoglycaemia [10]. Similarly, pharmacological or genetic manipulation of AMPK in neurons or the VMH is able to increase or decrease the counterregulatory response to hypoglycaemia [5, 9]. Discussion of other transporters, membrane channels or enzymes that contribute to glucose sensing, such as sodium–glucose cotransporters (SGLTs), transient receptor potential channels, Na^+^/K^+^ ATPase, K^+^ channels and nitric oxide synthase (NOS), are beyond the scope of this review, but interested readers are referred to a recent very detailed review of this area by Stanley et al [8]. Sherwin proposed that it was through the key signalling steps described above that a falling glucose level led to suppression of glucose-excited gamma aminobutyric acid (GABA)-inhibitory neurons, and activation of glutamatergic glucose-inhibited neurons, leading to progressive activation of the downstream counterregulatory response.

This hypothesis subsequently served as a model for examining the consequences of recurrent hypoglycaemia on glucose-sensing neurons. Electrophysiological studies demonstrated that recurrent hypoglycaemia resulted in a left-shift in glucose-excited neurons so that they did not hyperpolarise until glucose levels fell further [11]. This was consistent with reports of increased hypothalamic hexokinase activity following recurrent hypoglycaemia [12], implying that glucose-excited neurons were better able to maintain intercellular ATP:AMP ratios during subsequent hypoglycaemia and, hence, maintain GABAergic tone in the VMH [13]. A likely candidate mechanism for this was an increase in glycolytic flux in neurons. Rodent studies had shown that recurrent hypoglycaemia led to increased glucose transport activity at the blood–brain barrier (BBB) via an increase in total BBB GLUT1 and an increased transporter concentration at the luminal surface [14]. Similarly, in vivo microdialysis of brain extracellular fluid found higher glucose levels in rodents exposed to recurrent hypoglycaemia compared with control animals [15]. However, human studies using magnetic resonance spectroscopy or positron emission tomography (PET) have produced conflicting data about the effects of recurrent hypoglycaemia on cerebral glucose uptake and metabolism. This may reflect regional variation in glucose metabolism, or even differences in how neurons and glial cells individually respond to recurrent hypoglycaemia [8, 16, 17].

Alternatively, recurrent hypoglycaemia may induce cellular adaptations that allow lactate or ketones to be used as alternate fuels [8, 17]. Lactate or ketone infusions suppress counterregulation to systemic hypoglycaemia in humans [8], and lactate transport and lactate metabolism by the brain are thought to be increased in both humans [18, 19] and rodents [20] who have been exposed to recurrent hypoglycaemia. Initial research also suggested that recurrent hypoglycaemia led to increased astrocytic glycogen storage (supercompensation), which could provide additional lactate during subsequent hypoglycaemia, although these findings were not replicated in later studies [8]. Lactate might act to modulate the counterregulatory response to hypoglycaemia via suppression of AMPK [21] and increased GABA release [22]. However, other studies in humans [23] and rodents [20] suggest that lactate may be insufficient to support metabolism during hypoglycaemia and might instead act as a regulator of glucose metabolism, indicating that the alternate fuel hypothesis cannot fully explain how the brain adapts to recurrent hypoglycaemia.

**Fig. 1:**
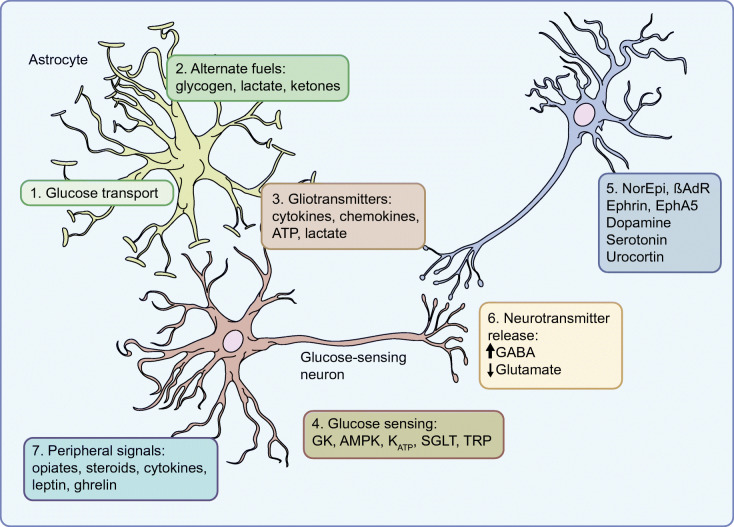
Potential mechanisms of cerebral adaptation to recurrent hypoglycaemia. This figure highlights some of the major mechanisms that have been shown to contribute to the suppression of counterregulatory responses that follows recurrent hypoglycaemia. In particular, research has highlighted potential roles for: (1) increased glucose transport; (2) increased use of alternate fuels; (3) gliotransmitters, such as cytokines; (4) increased phosphorylation and metabolism of glucose; (5) external neuronal modulators; (6) alterations in neurotransmitter release; and (7) peripheral signalling molecules, such as glucocorticoids. βAdR, β-adrenergic receptor; EphA5, ephrin receptor A5; GK, glucokinase; NorEpi, noradrenaline (norepinephrine); SGLT, sodium–glucose cotransporters; TRP, transient receptor potential channels. This figure is available as part of a downloadable slideset

In addition to changes in cellular fuel transport and metabolism in glucose-sensing neurons, a number of other factors have been proposed that could contribute, at least in part, to the development of impaired hypoglycaemia awareness (Fig. 1). External neuronal modulators, such as opioids, serotonin, steroids, cytokines or urocortin, have all been shown to modulate the counterregulatory response to insulin-induced hypoglycaemia and can affect changes in neurotransmitter synthesis or release, or changes in synaptic structure [2, 8]. More recently, it has also been proposed that the whole-organism response to recurrent hypoglycaemia represents a form of adaptive memory, referred to as ‘habituation’ [2]. This concept was tested by using a single challenge of high-intensity exercise as a dishabituatory stimulus to restore hypoglycaemic responses in rodents [24] and humans [25] with defective counterregulation. Stable isotope labelling with amino acids in cell culture (SILAC) mass spectroscopy identified a potential role for exercise-induced brain-derived neurotrophic factor (BDNF) in restoring hypothalamic glutaminergic transmission.

**Fig. 2:**
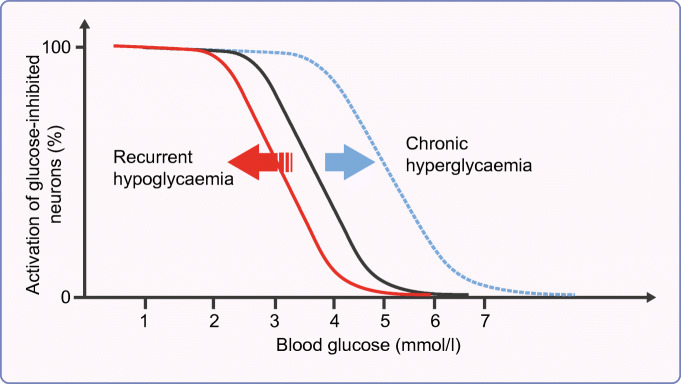
Shifting thresholds for hypoglycaemia detection. In this hypothetical model, in people without diabetes (represented by the black line), a progressive fall in blood glucose leads to gradual suppression of glucose-excited neuronal activity and an associated increase in glucose-inhibited neuronal activity, up to a maximum response. Recurrent (or chronic) hypoglycaemia leads to a left shift of this response curve (red line), while chronic hyperglycaemia leads to a right shift (blue line), both altering the threshold for initiating a counterregulatory hormone response to subsequent hypoglycaemia. Figure adapted from [2]. This figure is available as part of a downloadable slideset

In summary, recurrent hypoglycaemia may directly or indirectly lead to a series of adaptations in specialised glucose-sensing cells throughout the body, the net effect of which is to reduce the responsiveness of these cells to subsequent hypoglycaemia (a ‘left-shift’ in the counterregulatory response to subsequent hypoglycaemia; Fig. 2). Although this is considered an adaptive response, likely to be a mechanism to ensure the cells remain resilient to future periods of energy deprivation, this proves ‘maladaptive’ in diabetes, largely because of the inability to switch off exogenous insulin that is being released continuously from a subcutaneous depot, but also because of a hypoglycaemia-specific defect in alpha cell-derived glucagon release that is present in all people with type 1 diabetes after a few years of diagnosis and in some people with long-duration type 2 diabetes [9]. As a consequence, recurrent hypoglycaemia in diabetes leads both to impaired awareness of hypoglycaemia and a greatly increased risk of severe hypoglycaemia.

## Recurrent hypoglycaemia and the ‘pathobiology of diabetes complications’

The first part of this review described how recurrent hypoglycaemia induces a series of cellular adaptations in key glucose-sensing neurons that ultimately leads to their reduced responsiveness to low glucose levels. This effect is not specific to glucose-sensing neurons because the impact of recurrent hypoglycaemia is seen on cognitive functions and emotion, indicating cells in other brain regions are also affected [2]. This raises the question as to what the long-term effects of recurrent hypoglycaemia may be with regards to other aspects of brain function. Interestingly, while for many people with type 1 and 2 diabetes strict avoidance of hypoglycaemia can restore hypoglycaemia awareness [2], there remains a cohort of individuals who have complete and irreversible loss of awareness, which suggests sustained tissue damage.

Epidemiological research on the long-term impact of hypoglycaemia on the brain is limited, largely because most studies are only able to examine the association between severe hypoglycaemia and cognitive performance or brain structure. Moreover, most cases of severe hypoglycaemia do not result in hospitalisation or even ambulance call-out and, so, are under-recorded in healthcare databases, and the vast majority of hypoglycaemic episodes are not severe in nature. That being said, the various meta-analyses examining the impact of severe hypoglycaemia on brain structure and function indicate a small negative effect on a number of different cognitive domains, especially with early-onset type 1 diabetes [26, 27]. There is also evidence of structural changes in the brain in adults with type 1 diabetes who have impaired awareness of hypoglycaemia [28].

The brain is considered especially vulnerable to hypoglycaemia. This is due to its high metabolic demand, reliance on glucose as a primary fuel and minimal fuel stores. Rodent studies have demonstrated that severe hypoglycaemia results in significant brain damage through a number of mechanisms, including activation of neuronal glutamate receptors, production of reactive oxygen species (ROS), neuronal zinc release, activation of poly(ADP-ribose) polymerase–1, and mitochondrial permeability transition, with oxidative DNA damage thought to be the critical endpoint of hypoglycaemic stress (e.g., [29–31]). However, the degree of hypoglycaemia induced in these studies is extreme (usually <1.0 mmol/l glucose and sufficient to produce an isoelectric EEG) and, fortunately, this is seen rarely in humans with diabetes, albeit with an equally poor outcome.

A number of recent studies have examined the impact of recurrent moderate (2.5–3.5 mmol/l blood glucose) hypoglycaemia over a period of 2–4 weeks in rodent models of type 1 diabetes [32–34]. Recurrent hypoglycaemia was shown to exacerbate impairments in memory function that resulted from chronic hyperglycaemia [32, 34]. All three of these studies reported similar findings on examination of hippocampal tissue; recurrent hypoglycaemia in diabetic, but not non-diabetic, rodents resulted in disruptions of mitochondrial structure, dynamics and membrane potential, as well as alterations in mitochondrial energy metabolism. Evidence of increased oxidative stress and oxidative damage was also present, as well as increased ROS production, reduced antioxidant activity and increased inflammation [32–34]. There was also evidence of changes in synaptic morphology and reduced synaptic marker proteins [32–34]. Interestingly, recurrent moderate hypoglycaemia in non-diabetic rodents does not result in cognitive impairment or hippocampal damage [34] and, in a long-term study, it actually enhanced cognitive performance [35]. This implies that recurrent moderate hypoglycaemia is associated with oxidative damage only in the context of diabetes.

**Fig. 3:**
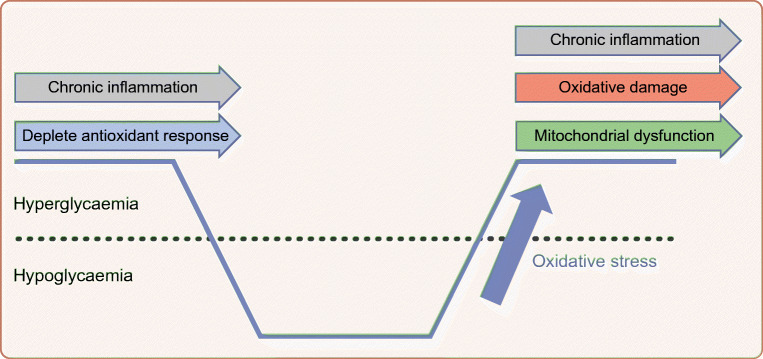
Impact of glycaemic variability, hypoglycaemia and hypoglycaemia recovery on the brain. Chronic hyperglycaemia depletes host antioxidant responses. Glucose recovery from hypoglycaemia represents a marked oxidative stress, which may overwhelm the capacity of vulnerable brain regions to respond to this, leading to mitochondrial dysfunction, oxidative damage and an exacerbation of the chronic inflammatory state that is associated with chronic hyperglycaemia. This figure is available as part of a downloadable slideset

Chronic hyperglycaemia is well recognised as having profound effects on many cell types. As described by Michael Brownlee in his 2004 Banting Lecture, the ‘Pathobiology of diabetes complications: a unifying mechanism’, hyperglycaemia leads to an overproduction of mitochondrial superoxide, resulting in protein kinase c (PKC) activation, increased advanced glycation end-products (AGE), increased hexosamine pathway activity and increased flux through the polyol pathway, leading to cellular damage and inflammation [36]. This chronic stimulation impairs cellular antioxidant responses; people with diabetes have reduced erythrocyte super oxide dismutase (SOD) [37, 38] and reduced total free-radical trapping capacity [39]. Chronic hyperglycaemia, therefore, induces chronic oxidative stress/inflammation and depletes host antioxidant defence mechanisms. Moreover, hypoglycaemia is both a proinflammatory stimulus and induces oxidative stress [40]. Therefore, the ability of cells to cope with hypoglycaemia-induced oxidative stress may be impaired in diabetes. Consistent with this, severe hypoglycaemia in chronically hyperglycaemic rodents induces more neuronal damage than in non-diabetic rodents [30], and diabetes acts synergistically with recurrent hypoglycaemia to impair mitochondrial function and induce oxidative damage in rodents [32–34]. Furthermore, hypoglycaemia-induced oxidative stress and neuronal death have been shown to occur primarily in the recovery period, during glucose reperfusion, and superoxide production and neuronal death increased with increasing glucose concentrations during the reperfusion period [41]. Based on these findings (summarised in Fig. 3) it is possible to speculate that the consequences of hypoglycaemia in vulnerable brain regions, such as the hippocampus are dependent not only on the extent of hypoglycaemia, but also the quality of prior glycaemic control and the extent of rebound hyperglycaemia on recovery from the hypoglycaemic episode.

## Summary

The value of insulin in the management of diabetes and the evidence in support of intensive insulin therapy targeting near-normalisation of glycaemic control to minimise the micro- and macrovascular complications of diabetes is overwhelming. However, hypoglycaemia remains a relatively common adverse effect of insulin therapy that has consequences for the individual and their carers. Asides from the immediate cognitive and emotional impacts of acute hypoglycaemia, impaired hypoglycaemia awareness is a consequence of repeated exposure to hypoglycaemia that carries a high risk for severe hypoglycaemia. Epidemiological and pre-clinical research also indicates that recurrent hypoglycaemia may exacerbate chronic hyperglycaemia-induced increases in oxidative stress and inflammation, leading, in particular, to damage in vulnerable brain regions and accelerated cognitive decline.

There remain many unanswered questions that hopefully future research will be able to shed light on. For instance, do the effects of recurrent hypoglycaemia on specialised glucose-sensing neurons occur through a single signalling defect or multiple pathways given the complexity of the hypoglycaemic response? Moreover, is there actually a ‘defect’ in sensing or are glucose-sensing neurons just less responsive to low glucose? If the latter, do the major changes specifically occur in the specialised neuron or in the periphery (e.g., adrenal gland)? It is also uncertain whether these effects are reversible in all people through strict hypoglycaemic avoidance or whether, in some, these may prove irreversible as a consequence of long-term damage to critical components of this homeostatic defence mechanism.

When we consider other consequences of recurrent hypoglycaemia on the brain, we also need clarity on the actual level of hypoglycaemia that is clinically significant, which may or may not be 3.0 mmol/l as recently proposed by the International Hypoglycaemia Study Group [42]. It is also possible that recurrent hypoglycaemia has consequences in other metabolically active tissues, such as the heart and kidney, whereby the interaction between prior glycaemic control, hypoglycaemia and the degree of rebound hyperglycaemia may explain why increased glycaemic variability is considered a risk factor for complications in multiple organ systems [43].

## Supplementary Information

Slideset of figures (PPTX 503 kb)

## Abbreviations

AMPK: AMP-activated protein kinase
BBB: Blood–brain barrier
GABA: Gamma aminobutyric acid
K_ATP_: ATP-sensitive potassium
ROS: Reactive oxygen species
VMH: Ventromedial hypothalamus
