# Supplementary material for: Consequences of recurrent hypoglycaemia on brain function in diabetes
Source: Diabetologia. 2021 Mar 18;64(5):971–7. doi: 10.1007/s00125-020-05369-0 (PMC8012314; doi:10.1007/s00125-020-05369-0)
Supplement: Supplementary file 1 — (PPTX 503 kb) [file 125_2020_5369_MOESM1_ESM.pptx]

## Slide 1
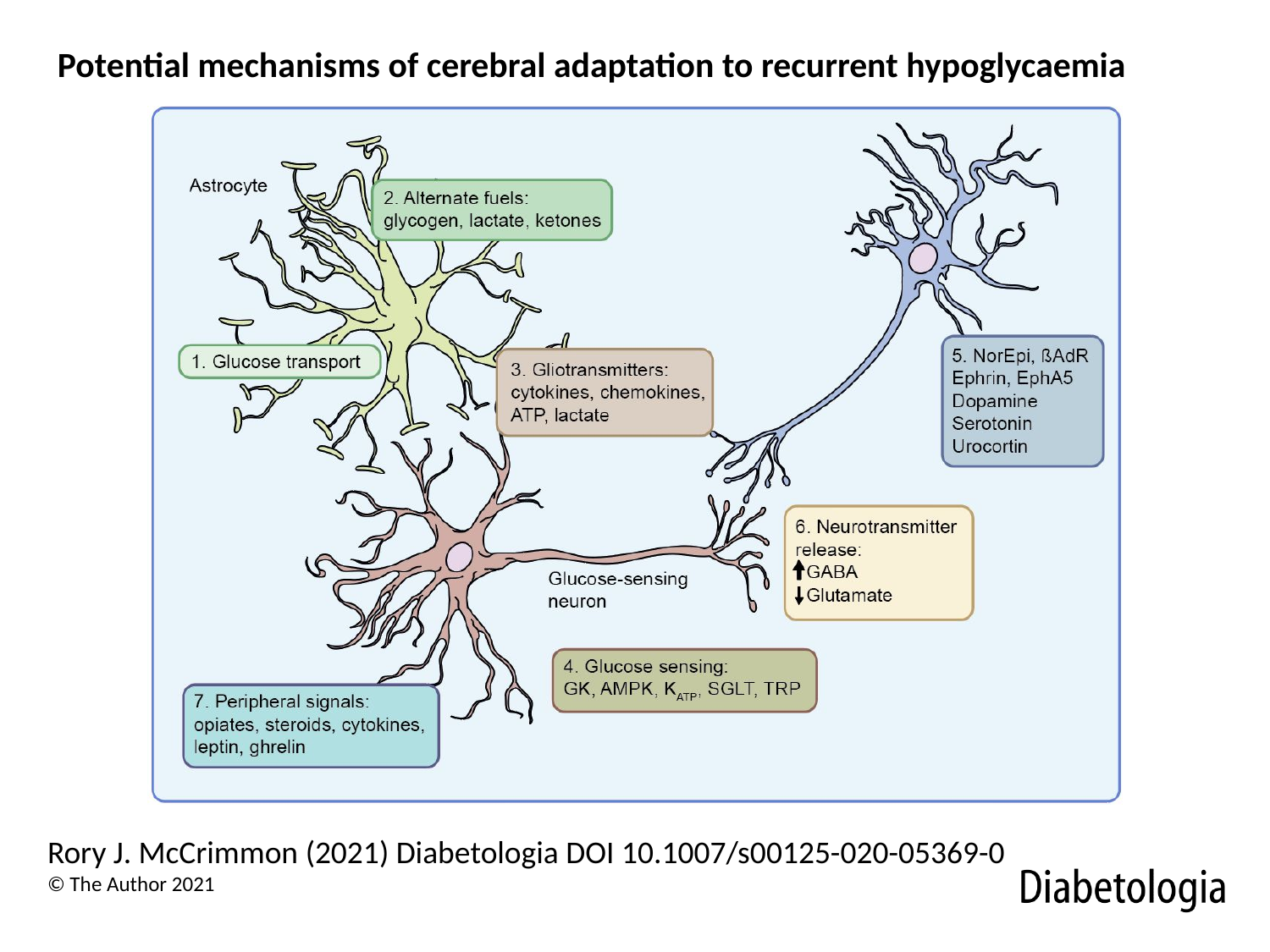

Potential mechanisms of cerebral adaptation to recurrent hypoglycaemia
Rory J. McCrimmon (2021) Diabetologia DOI 10.1007/s00125-020-05369-0
© The Author 2021

## Slide 2
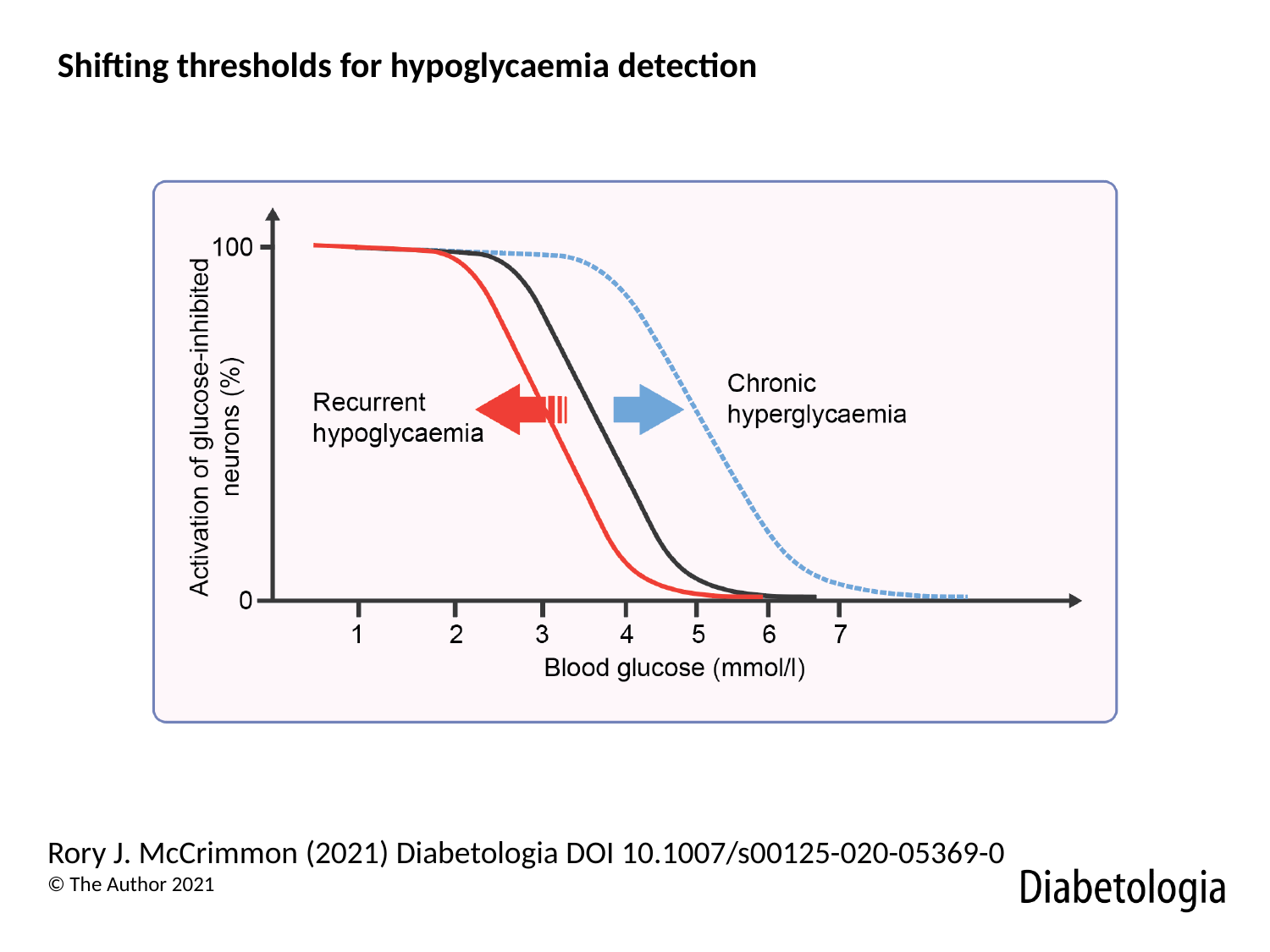

Shifting thresholds for hypoglycaemia detection
Rory J. McCrimmon (2021) Diabetologia DOI 10.1007/s00125-020-05369-0
© The Author 2021

## Slide 3
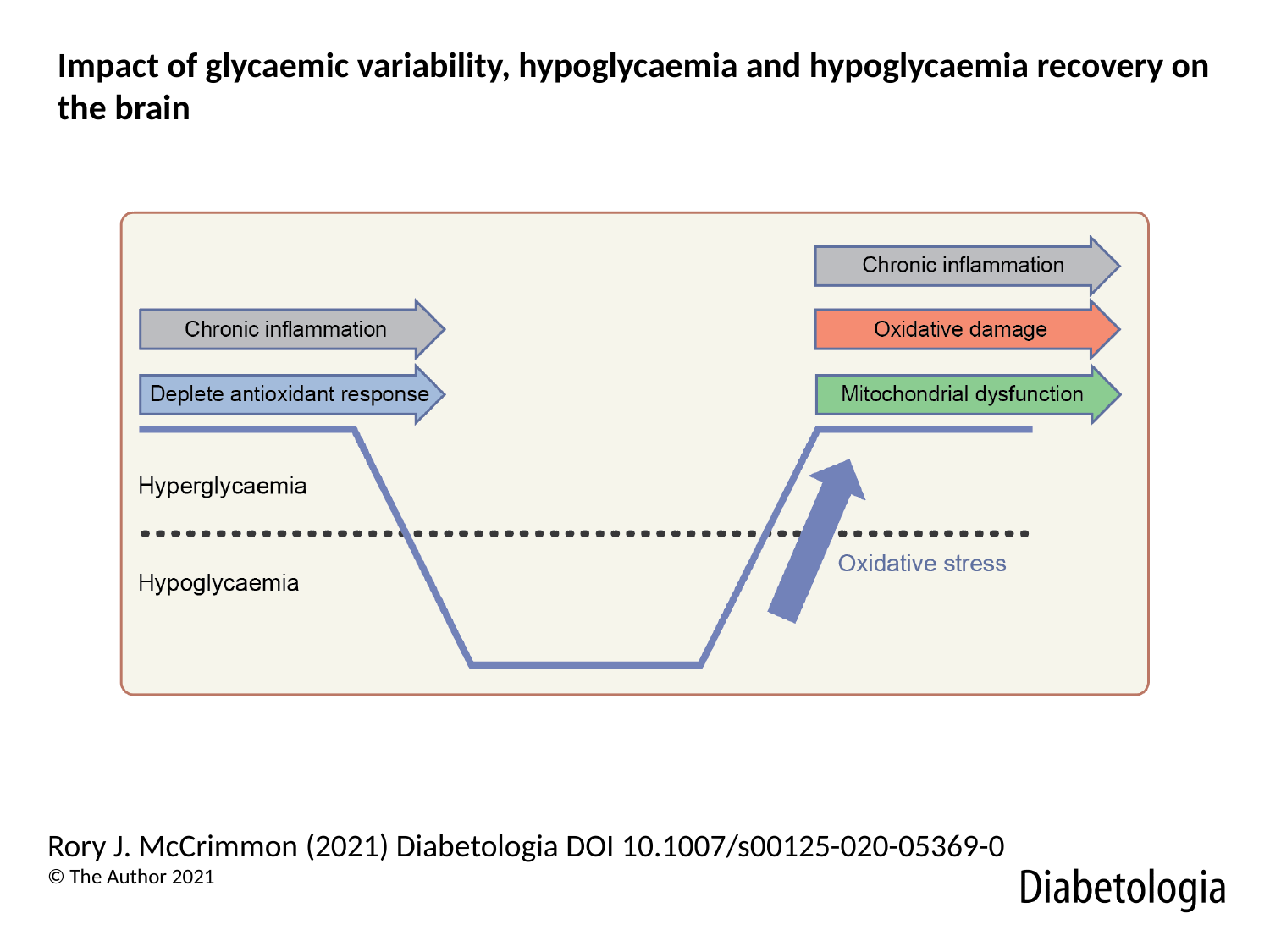

Impact of glycaemic variability, hypoglycaemia and hypoglycaemia recovery on the brain
Rory J. McCrimmon (2021) Diabetologia DOI 10.1007/s00125-020-05369-0
© The Author 2021
